# Trends in SARS-CoV-2 infection and vaccination in school staff, students and their household members from 2020 to 2022 in Wales, UK: an electronic cohort study

**DOI:** 10.1177/01410768231181268

**Published:** 2023-06-22

**Authors:** Emily Lowthian, Hoda Abbasizanjani, Stuart Bedston, Ashley Akbari, Laura Cowley, Richard Fry, Rhiannon K Owen, Joe Hollinghurst, Igor Rudan, Jillian Beggs, Emily Marchant, Fatemeh Torabi, Simon de Lusignan, Tom Crick, Graham Moore, Aziz Sheikh, Ronan A Lyons

**Affiliations:** 1 Population Data Science, Swansea University Medical School, 7759 Faculty of Medicine, Health and Life Science, Swansea University, SA2 8PP, UK; 2 Department of Education & Childhood Studies, School of Social Sciences, Faculty of Humanities and Social Sciences, Swansea University, SA2 8PP, UK; 3 Centre for Global Health, Usher Institute, The University of Edinburgh, Edinburgh, EH8 9AG, UK; 4 Usher Institute, The University of Edinburgh, Edinburgh, EH8 9AG, UK; 5 Nuffield Department of Primary Care Health Sciences, University of Oxford, OX2 6GG, UK; 6 DECIPHer, 2112 SPARK, Cardiff University, Cardiff, CF24 4HQ, UK; These authors contributed equally to this work.

**Keywords:** COVID-19, schools, social restrictions, pandemic, population health

## Abstract

**Objectives:**

We investigated SARS-CoV-2 infection trends, risk of SARS-CoV-2 infection and COVID-19 vaccination uptake among school staff, students and their household members in Wales, UK.

**Design:**

Seven-day average of SARS-CoV-2 infections and polymerase chain reaction tests per 1000 people daily, cumulative incidence of COVID-19 vaccination uptake and multi-level Poisson models with time-varying covariates.

**Setting:**

National electronic cohort between September 2020 and May 2022 when several variants were predominant in the UK (Alpha, Delta and Omicron).

**Participants:**

School students aged 4 to 10/11 years (primary school and younger middle school, *n* = 238,163), and 11 to 15/16 years (secondary school and older middle school, *n* = 182,775), school staff in Wales (*n* = 47,963) and the household members of students and staff (*n* = 697,659).

**Main outcome measures:**

SARS-CoV-2 infection and COVID-19 vaccination uptake.

**Results:**

School students had a sustained period of high infection rates compared with household members after August 2021. Primary schedule vaccination uptake was highest among staff (96.3%) but lower for household members (72.2%), secondary and older middle school students (59.8%), and primary and younger middle school students (3.3%). Multi-level Poisson models showed that vaccination was associated with a lower risk of SARS-CoV-2 infection. The Delta variant posed a greater infection risk for students than the Alpha variant. However, Omicron was a larger risk for staff and household members.

**Conclusions:**

Public health bodies should be informed of the protection COVID-19 vaccines afford, with more research being required for younger populations. Furthermore, schools require additional support in managing new, highly transmissible variants. Further research should examine the mechanisms between child deprivation and SARS-CoV-2 infection.

## Introduction

The world has changed considerably since the emergence of SARS-CoV-2, the virus that causes COVID-19. Most countries worldwide closed educational institutions, such as schools, with the intention of minimising the spread of SARS-CoV-2. In the UK, specifically, most children faced over 6 months of home learning, and some children had fewer resources, equipment and lived in potentially unsafe conditions.^1–3^ Over the COVID-19 pandemic, debates have unfolded surrounding infection, transmission, vaccination and secondary harms.

The susceptibility of children and young people to COVID-19^4^ and the risk of severe outcomes across different variants have been a key focus.^5^ One of the first systematic reviews found that the initial SARS-CoV-2 variant was related to milder symptoms and fewer infections in children^6^; however, later studies captured greater infection rates^7^; some studies have reported more adverse reactions in children following the Omicron wave.^8^ In terms of the education workforce, research is inconsistent as to whether SARS-CoV-2 is more common in school staff than in the community^9^ or students.^10^ The school’s role in transmission points to community rates being the main predictor of infections,^7,10^ with schools being unlikely to play a critical role in transmission^9,11^; however, most studies focus on the initial and Alpha variant periods, but newer research suggests greater infections due to the Delta variant.^12^ Alongside the primary effects of COVID-19, the secondary harms include the negative impact on the mental health and wellbeing of children^13^ and the educational workforce.^14^

To reduce the severity of COVID-19, vaccines were rapidly developed to reduce the risk of hospitalisation or death. In the UK, school staff advocated to be considered a priority; however, this was unsuccessful, and priority was based on age and clinical vulnerability.^15^ Vaccination was made available for students aged 16–17 years in August 2021 and in September 2021 for those aged 12–15 years, but not until February 2022 for 5–11 year olds.^15^ Current debates now surround whether the potential risk of vaccination outweighs the small benefit vaccination offers for children.^16^

Given uncertainties in infection patterns amongst children, educational staff, and their households, we developed a national cohort of linked data to report on the following: (i) the trends in infection over 19 months in school staff, students and their household members; (ii) vaccination uptake for school staff, students, and their household members, and (iii) examine the association between SARS-CoV-2 infection and demographic characteristics, dominant variant and vaccination across school staff, students, and their household members.

## Methods

### Study design and participants

**Figure 1.**
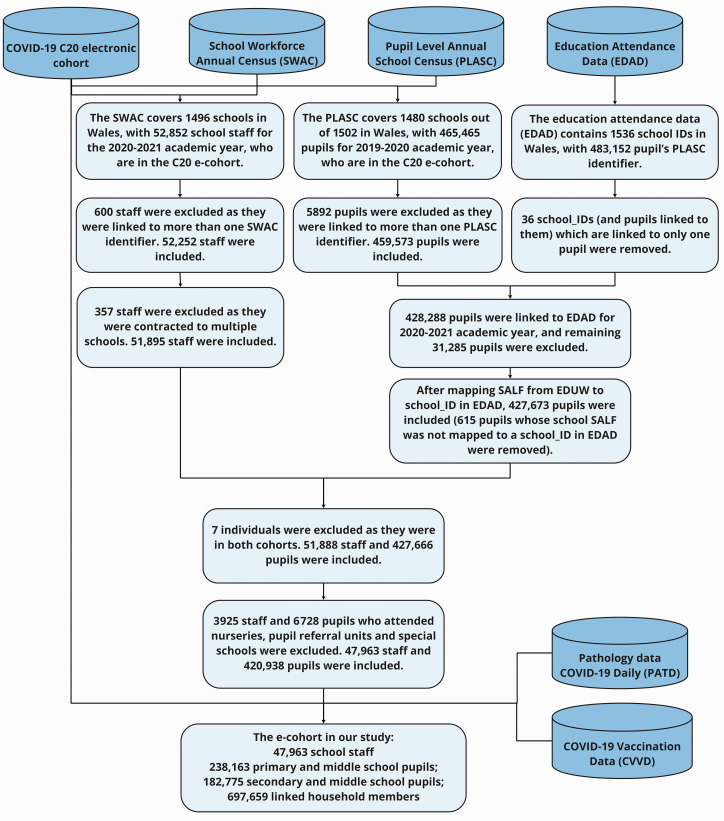
Health and administrative education data linkage.

We created a national e-cohort of school students, staff and linked household members (details outlined in Thompson et al.^11^) for both students and staff in Wales (Figure 1). We used anonymised linked population-scale, individual-level data held within the Secure Anonymised Information Linkage (SAIL) Databank at Swansea University to create the e-cohort at an individual and household level.^17–19^ The data in SAIL are de-identified using multiple encryptions by different organisations. All proposals to use anonymised data in SAIL are assessed by an independent Information Governance Review Panel (IGRP).

### Procedure

Our population spine was the Welsh COVID-19 e-cohort, termed C20,^20^ which consists of all people alive and known to the NHS in Wales since 1 January 2020. We linked the Welsh Government’s School Workforce Annual Census for the 2020/2021 academic year, which details all individuals who work in a publicly funded school,^21^ covering 1496 out of 1502 schools in Wales, and the Pupil Level Annual School Census (PLASC) for the 2019/2020 academic year, which includes annual returns on 1480 out of 1502 schools.^22^ We also linked Education Attendance Data for the 2020/2021 academic year to PLASC to confirm their educational settings for this academic year.

We linked staff and students to all other individuals within the same household using Residential Anonymised Linkage Field (RALF, as in Johnson et al.^23^) in Wales, available in the C20 e-cohort. Our e-cohort used students, staff and linked household members grouped into educational settings using a unique School Anonymised Linking Field (SALF, as in Thompson et al.^11^). We also linked the cohort to COVID-19 antigen polymerase chain reaction (PCR) testing data to identify confirmed cases of SARS-CoV-2 infection (Figure 1). We followed participants from 3 September 2020 to 31 May 2022. We removed staff members contracted to multiple schools as it was not possible to determine durations within each school. To define the type of school, we combined primary and middle school students (who were aged >3 and <11 years), and secondary and middle school students (aged >10 and <17 years) as the middle school group was small (<5%). We excluded students and teachers who attended nurseries, pupil referral units and special schools. For SARS-CoV-2 infection, the dates of the first to fourth positive PCR COVID-19 antigen tests for school students, school staff and their linked household members were identified (between 28 February 2020 and 31 May 2022) with a 90-day clearance before a subsequent positive test.

### Ethics

We accessed the data following approvals from the SAIL independent IGRP approval, which permits access to anonymised data via the SAIL Databank.

### Statistical analysis

We examined the count of daily infections from 3 September 2020 until 31 May 2022; results for the number of tests between 1 and 10 a day were capped as 5 due to disclosure control processes. We show key dates, including periods of the dominant variant of SARS-CoV-2, school restrictions, vaccination delivery and social restrictions, with ‘Alert level 4’ being the highest restrictions and ‘Alert level 0’ depicting low restrictions. To examine the uptake of the COVID-19 vaccination, we calculated the cumulative incidence over time and plotted this for school students, staff and their household members. To model the risk of the first SARS-CoV-2 infection, we used Poisson multi-level models (MLM) in the glmmTMB package in R,^24^ with vaccination and dominant variant periods as time-varying covariates and duration of periods as an offset. The hierarchical structure was school and household identifier (separate effects) for school staff and students. For household members, we used Unitary Authority (a small area of governance) and household identifier (nested effects); we could not use a smaller area due to multi-collinearity with our household deprivation measure. We adjusted the Poisson MLM for fixed-effects including sex, free school meal (FSM) entitlement – a measure of low income, Special Educational Needs (SEN), also known as Additional Learning Needs and deprivation quintiles (based on the Welsh Index of Multiple Deprivation, WIMD version 2019).

## Results

Table 1 shows that sex was equal for all groups except the school staff force (84% female). Nearly a quarter of students and household members were from the most deprived quintile, whereas 26% of school staff were from the least deprived quintile. Around one-third of primary and younger middle school students had documented SARS-CoV-2 infection at least once (31%), as did 36% of secondary and older middle school students, 41% of staff and 29% of household members. Multiple infections were low in prevalence (<2%) and fourth infections are not shown due to small counts, but are used in the trend analysis.

**Table 1.** Background characteristics of school students, staff and household members.

	Primary and middle school students(*n* = 238,163)	Secondary and middle school students(*n* = 182,775)	School staff(*n* = 47,963)	Household members (*n* = 697,659)
Age				
Mean	6.41	12.8	41.6	35.3
Median	6	13	42	37
S.D.	2.08	1.84	11.2	16.6
Sex				
Male	121,272 (51%)	91,863 (50%)	7624 (16%)	339,668 (49%)
Female	116,891 (49%)	90,912 (50%)	40,339 (84%)	357,991 (51%)
Welsh index of multiple deprivation				
Most deprived	58,068 (24%)	40,337 (22%)	5347 (11%)	165,383 (24%)
Second	47,295 (20%)	35,281 (19%)	7975 (17%)	142,098 (20%)
Third	40,919 (17%)	31,912 (18%)	9118 (19%)	131,030 (19%)
Fourth	38,952 (16%)	32,085 (18%)	10,416 (22%)	126,942 (18%)
Least deprived	39,280 (17%)	35,249 (19%)	12,459 (26%)	131,973 (19%)
School type				
Primary	231,142 (97%)	–	29,362 (61%)	–
Middle	7021 (3%)	12,242 (7%)	2,162 (5%)	–
Secondary	–	170,533 (93%)	16,439 (34%)	–
Free school meals	60,627 (26%)	38,668 (21%)	–	–
Special educational needs	42,636 (18%)	40,596 (22%)		
SARS-CoV-2 infection				
Once	73,259 (31%)	65,562 (36%)	19,613 (41%)	200,881 (29%)
Twice	2852 (1%)	2484 (1%)	1174 (2%)	12,353 (2%)
Three	12 (>1%)	17 (>1%)	13 (>1%)	266 (>1%)
Had PCR test	180,409 (76%)	134,814 (74%)	41,174 (86%)	484,168 (69%)

**Table 2.** Poisson multi-level models estimating the count of SARS-CoV-2 infection for primary/middle and secondary/middle school students, school staff and household members; vaccination and dominant variant as time-varying covariates.

	Primary/middle school students	Secondary/middle school students	School staff	Household members
*n*	224,422	174,767	45,242	675,754
Missing (%)	5.8	4.4	5.7	3.1
Household groups	164,316	136,219	42,820	269014
School/LA groups	1239	204	1425	22
Age	1.17 (1.16–1.17)	1.01 (1.00–1.01)	0.99 (0.98–0.99)	1.00 (1.00–1.00)
Sex (female)	1.02 (1.00–1.04)	1.17 (1.15–1.19)	1.03 (0.98–1.09)	1.37 (1.36–1.38)
WIMD (reference = most deprived)				
2	1.09 (1.05–1.12)	1.07 (1.03–1.10)	0.86 (0.80–0.93)	1.06 (1.04–1.08)
3	1.11 (1.08–1.15)	1.05 (1.02–1.09)	0.79 (0.73–0.85)	0.97 (0.95–0.99)
4	1.20 (1.16–1.24)	1.06 (1.02–1.09)	0.79 (0.73–0.85)	0.96 (0.93–0.98)
Least deprived	1.28 (1.24–1.33)	1.14 (1.10–1.18)	0.87 (0.81–0.94)	1.02 (1.00–1.04)
Vaccination (reference = unvaccinated)				
First dose	0.42 (0.38–0.45)	1.01 (0.99–1.04)	0.23 (0.21–0.26)	0.85 (0.84–0.87)
Second dose	0.13 (0.10–0.17)	0.36 (0.35–0.38)	0.60 (0.55–0.65)	1.38 (1.36–1.40)
Booster dose	0.18 (0.02–1.32)	0.26 (0.24–0.29)	0.23 (0.21–0.25)	0.91 (0.89–0.92)
Dominant variant period (reference = alpha)				
Delta	17.62 (17.17–18.08)	14.72 (14.36–15.08)	7.91 (7.32–8.55)	3.19 (3.15–3.24)
Omicron	15.26 (14.78–15.75)	10.99 (10.61–11.38)	22.49 (20.51–24.65)	5.02 (4.94–5.10)
Student FSM	0.75 (0.73–0.77)	0.72 (0.70–0.74)	–	–
Student SEN	0.93 (0.90–0.95)	0.84 (0.82–0.86)	–	–

FSM: free school meal; SEN: Special Educational Needs.

### Overall trends

**Figure 2.**
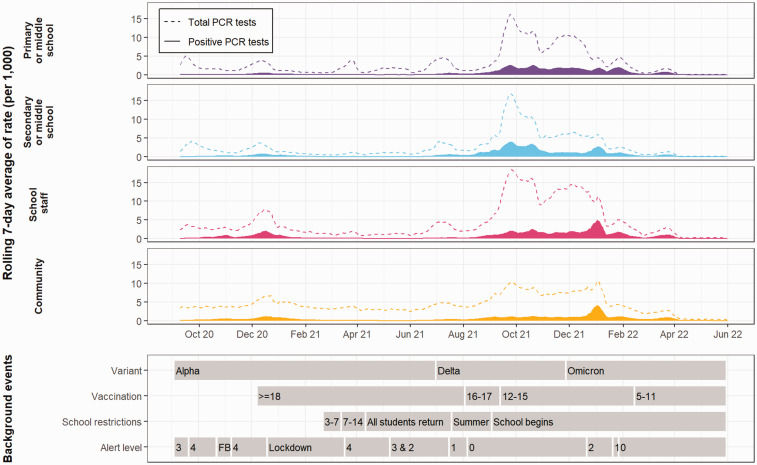
Crude rate of infections and PCR tests per 1000 (rolling 7-day average) from September 2020 to May 2022 for primary and younger middle (purple) and secondary and older middle school students (blue), school staff (pink) and household members (yellow). Dashed lines = rate of PCR tests, Solid fill = rate of positive PCR tests. Events are shown for dominant variant, vaccination programmes, school restrictions and social restrictions, whereby higher alert levels indicate greater restrictions (FB = firebreak). Note: PCR testing for the public ends on 31 March 2022.

Annotated trends are shown for school students, staff and household members (Figure 2). All groups show a wave of infection in late 2020 into early 2021, which was higher for school staff and household member groups. Over this period, the wave of infections prompted a ‘Firebreak’ (FB) in Wales, whereby stronger social restrictions were in place between 23 October 2020 to 9 November 2020 and a later lockdown on 19 December 2020. The first 6 months of 2021 show a decrease in infections, but they began to rise when students finished school for summer, which coincides with the removal of social restrictions, termed as ‘Alert Level 0’, and the emergence of the Delta variant. From August 2021 onwards, there was a large wave of infections, and a larger proportion of school students had SARS-CoV-2 between September 2021 and December 2021 compared with school staff or household members. The rise in infections remained stable until the emergence of the Omicron variant, when all groups showed an increase in infections; less of a peak was observed for primary and younger middle school students. Infections began to decrease after January 2022, following increased social restrictions, but once these were removed, infections began to increase up to the end of March 2022; PCR testing ended on 31 March 2022 for the public. To view the rate of PCR tests and positive PCR tests per 1000 in the periods of the FB and lockdown in detail, when increased social restrictions were in place, see the Supplementary Material.

### Vaccination uptake

**Figure 3.**
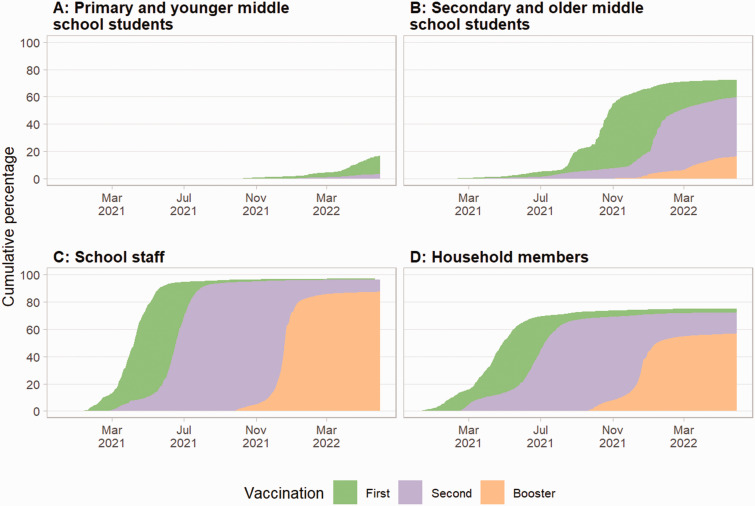
Cumulative percentage of vaccination uptake from 3 September 2020 to 31 May 2022 for (a) primary and middle and (b) secondary and middle school students, (c) school staff and (d) the household members. Green indicates first dose, purple second and orange booster dose.

Figure 3 shows the uptake of COVID-19 vaccination. Primary and younger middle school students (Figure 3(a)) show the lowest uptake of vaccination, with 17.1% of the total population receiving a first dose, and 19.4% of these students received a second dose; few children received a booster dose, as vaccination began in February 2022. Overall, 72.4% of secondary and older middle school students have received a first dose, and of these, 82.5% have had a second dose, and 27.5% have received a booster dose. School staff had the highest uptake of vaccination, with 97.0% of the total population having a first dose and 99.3% of these having had a second dose, and 90.7% having had a booster dose. For household members, 75.2% of the population received a first dose, and of these, 96.1% had a second dose, but only 78.7% of these received a booster dose.

### Infection among school students, staff and their household members

Primary and younger middle school students showed that an increase in age posed a greater risk of SARS-CoV-2 (RR: 1.17, 95% CI: 1.16–1.17); likewise, girls were at a slightly higher risk (RR: 1.02, 95% CI: 1.00–1.04). The least deprived groups were at a higher risk of infection (RR: 1.28, 95% CI: 1.24–1.33); however, students who received FSM were less likely to have SARS-CoV-2 (RR: 0.75, 95% CI: 0.73–0.77), as were those who had SEN (RR: 0.93, 95% CI: 0.90–0.95). The first dose of the COVID-19 vaccine (RR: 0.42, 95% CI: 0.38–0.45) and second dose (RR: 0.13, 95% CI: 0.10–0.17) were negatively associated with infection; the booster dose had very few numbers and no association was found (RR: 0.18, 95% CI: 0.02–1.32). The Delta variant was associated with increased infection compared with Alpha (RR: 17.62, 95% CI: 17.17–18.08) as was Omicron (RR: 15.26, 95% CI: 14.78–15.75).

Secondary and older middle school students showed that increased age had a slightly greater risk of infection (RR: 1.01, 95% CI: 1.00–1.01). Girls (RR: 1.17, 95% CI: 1.15–1.19) and the least deprived groups (RR: 1.14, 95% CI: 1.10–1.18) had a higher risk. Students receiving FSM (RR: 0.72, 95% CI: 0.70–0.74) or with SEN (RR: 0.84, 95% CI: 0.82–0.86) were at lower risk. The first dose of vaccine (RR: 1.01, 95% CI: 0.99–1.04) was not associated with infection, but the second dose (RR: 0.36, 95% CI: 0.35–0.38) and booster dose (RR: 0.26, 95% CI: 0.24–0.29) were negatively associated. The Delta variant was positively associated with SARS-CoV-2 compared with Alpha (RR: 14.72, 95% CI: 14.36–15.08), as was Omicron (RR: 10.99, 95% CI: 10.61–11.38).

The model for school staff showed that as age increased by each year there was a slight decreased risk of SARS-CoV-2 infection (RR: 0.99, 95% CI: 0.98–0.99); sex showed no association (RR: 1.03, 95% CI: 0.98–1.09). Less deprived groups were less likely to be infected, particularly the third and fourth least deprived groups (RR: 0.79, 95% CI: 0.73–0.85). The first dose of vaccine (RR: 0.23, 95% CI: 0.21–0.26), second dose (RR: 0.60, 95% CI: 0.55–0.65) and booster dose (RR: 0.23, 95% CI: 0.21–0.25) were negatively associated with infection. The period of Delta variant dominance was associated with increased infection compared with the Alpha variant (RR: 7.91, 95% CI: 7.32–8.55), as was the period of Omicron dominance (RR: 22.49, 95% CI: 20.51–24.65).

For household members, age had no association with SARS-CoV-2 (RR: 1.00, 95% CI: 1.00–1.00) but females were more likely to be infected (RR: 1.37, 95% CI: 1.36–1.38). The second most deprived quintile was at a slightly higher risk of SARS-CoV-2 infection compared with the most deprived quintile (RR: 1.06, 95% CI: 1.04–1.08), as did the least deprived quintile (RR: 1.02, 95% CI: 1.00–1.04); the third and fourth quintiles showed a slight decrease. The first dose of vaccine (RR: 0.85, 95% CI: 0.84–0.87) and booster dose (RR: 0.91, 95% CI: 0.89–0.92) were negatively associated with infection, but the second dose was positively associated (RR: 1.38, 95% CI: 1.36–1.40). The Delta variant was positively associated with SARS-CoV-2 compared with the Alpha variant (RR: 3.19, 95% CI: 3.15–3.24), as was Omicron (RR: 5.02, 95% CI: 4.94–5.10). For estimates in full, see Table 2.

## Discussion

Our study presents multiple findings on SARS-CoV-2 infection and vaccination for school students, staff and their household members. All school students and staff had a stable period of higher infection compared with household members after August 2021, whereas before this, the rates were lower. The sustained period of infection for students in the remaining 6 months of 2021 is likely due to the Delta variant, as it was dominant in Wales until late December 2021.^15^ During the same period, Wales largely removed social restrictions, which led to a combination of increased social contacts with minimum prevention (e.g. masks). Whether the school environment provided increased opportunities for transmission cannot be confirmed here, but we do observe that the increase in infections for students began in July 2021 – after students finished school for the summer. Other studies that use data in pre-Delta and Omicron periods suggest that school is unlikely to be associated with high rates of transmission^9,11^; however, there appears to be limited research after this period. Statistics from Euro surveillance show a sharp increase in COVID-19 for school-aged children and their household members from August to October 2021, and theorise this was partially attributed to the Delta variant.^12^ In this period, students were unvaccinated and unlikely to have had prior infection compared with school staff and household members who may have had both, resulting in a lower rate of infection over time in the Delta period. However, staff and household members did show higher peaks of infection in the winter period, pointing to the risk of breakthrough infections via the Omicron variant.

We found that less deprived students were more likely to test positive for COVID-19, and the association was larger in effect size for children who received FSM, which captures children whose parents or carers are likely recipients of government financial support. We theorise that this association may be a proxy of child socialisation as children of more affluent families benefit from access to extra-curricular activities, such as after school clubs, classes or the swimming pool, whereas more deprived children may spend greater time at home. A recent study found that physical activity, out-of-school club participation and the ability to ride a bike were positively associated with COVID-19.^25^ Our data showed that children living in more deprived areas had a lower rate of testing compared with children living in the least deprived areas (see Supplementary Material). This may be reflective of potential concerns regarding absence from work as a result of child illness for more deprived communities compared with least deprived. Again, this study also found that girls were at a higher risk of SARS-CoV-2 infection, in line with our findings.^25^ They suggest that sex differences in time-use could be a factor, i.e. girls are more likely to spend time indoors rather than outdoors, contributing to a higher risk of infection.^25^ For household members and deprivation, a more complex trend appeared, whereby the second most deprived and fifth least deprived groups had a higher risk. Wider research suggests that COVID-19 mortality is higher in more deprived groups^26^; hence, the household members captured in our study may systematically differ to the general population. The risk of infection for less deprived school staff was lower compared with the most deprived quintile; this may be due to job role and the high transmission risk for lower paid staff (e.g. teaching assistants with close-contacts).

We also found that children who had SEN were less likely to test positive for COVID-19, which may coincide with the vulnerability of some children in this subgroup and the high concern around this by families.^27,28^ That being said, some parents were keen for their children with SEN to return to school to improve their routine and learning^27,28^; more research is required in this area to understand differing needs, risk of COVID-19 and testing consistency.

Vaccination rates were highest among school staff and household members and lower among school students, specifically primary and younger middle school students. Models revealed that vaccination had a negative association with subsequent SARS-CoV-2 infection, but effects were larger for school staff and students compared with their household members. We theorise that the stronger effects for students are likely due to recent vaccination and, subsequently, less time for waning of effectiveness. For staff, it may be due to the addition of preventative measures in the workplace and greater consistency in social mixing with students and others who were testing regularly as encouraged by the Welsh Government.^15^ The household members had the lowest effectiveness for vaccination and even a positive association for dose two. We attribute this to household members potentially receiving the vaccine earlier so greater waning, particularly given the distance between dose two and the booster, but also their likely increased social contacts from multiple settings, whereas students and staff socialised in ‘bubble-like’ settings.

The high rate of vaccination among teachers is encouraging and aligns with the previous campaign to be prioritised for vaccination in the UK, and wider views on critical national infrastructure in classrooms.^29^ While our research lends that they may have been at a greater risk of infection compared with other groups (as 41% were infected), other research shows that they were not at a higher risk for severe outcomes.^30^ Primary and younger middle school students’ uptake was the lowest among all groups but has only been approved for all children since February 2022^31^; children turning 5 years of age now are no longer automatically eligible, unless they are part of a high-risk group.^32^ While the 17.6% uptake may be considered low, the risk of severe outcomes among young children is low, and vaccination is likely to benefit adults via lower transmission.^16^ While this may be applicable to COVID-19 vaccines, public health bodies must ensure that accessible scientific information is available and widely communicated for other routine vaccinations such as MMR, as there has been a noted decrease in uptake which could cause an uprise in previously controlled diseases.^33^

While our study has several strengths, including almost complete coverage of students and staff in public schools for over 18 months, it also has several limitations. First, we did not have educational data for the 2021–2022 academic year, so our models assume that students and staff had the same educational characteristics; to ascertain this, we compared estimates, whereby we excluded students who would have left primary and secondary school in 2020–2021. We found that estimates were similar, so we retained these students in the original models. Second, we relied on PCR testing to confirm infection and some individuals will not have been tested. The Omicron period may have higher associations than estimated, as PCR testing was no longer freely accessible by the general public after March 2022. Lastly, the rate of school staff and student infections may be elevated due to the routine testing expected of these groups, as observed in Figure 2, rather than the variant or school setting itself.

To our knowledge, this is the first study to use a near complete school cohort for an extended period (19 months) to examine trends and associations with SARS-CoV-2 infection in school students, staff and their household members. We show that school staff and students had different patterns and prevalence of COVID-19 between September 2020 and May 2022 in Wales, UK. Statistical models indicated that the Delta and Omicron dominant periods posed an increased risk of SARS-CoV-2 infection for all groups, but to counter this, vaccination was associated with reduced risk of SARS-CoV-2 infection; we also found complex associations with demographic factors. From this, future strategies should acknowledge the protection vaccination affords for older school students and staff, while advancing their understanding of younger school children and vaccination. Further research should attempt to understand the relationship between sociodemographic factors, particularly deprivation, and SARS-CoV-2 infection. Lastly, more transmissible variants (i.e. Delta and Omicron) appeared to have a greater impact on school staff and students compared with household members, and schools may be vulnerable to future, more transmissible variants.

## Evidence before this study

We screened 1544 empirical studies using the terms (‘COVID-19’) OR (‘SARS-CoV-2’) AND (‘school*’) in the titles of articles via PubMed. The search was conducted on 24 August 2022, with articles not in English or published before 2020 excluded; 35 duplicates were removed. Thirty-five studies were included and focused on the prevalence of COVID-19 and vaccination uptake in school students or staff. Articles were excluded if they primarily examined: school closures, public health strategies, transmission or were simulation studies. Studies were mixed in terms of student incidence rates of SARS-CoV-2 in 2020; research from England, UK suggested low prevalence. However, studies showed a higher prevalence of SARS-CoV-2 in 2021, some associated with the Delta variant, particularly in European studies. Most studies suggested that SARS-CoV-2 incidence was similar between school staff and students, but this was not a consensus; studies were mixed when comparing incidence with household members or the community. Most studies did not find differences between school students’ age and COVID-19 incidence. Vaccination uptake varied largely across studies for school students and staff, with deprivation and vaccine hesitancy being associated with uptake. Vaccine effectiveness against SARS-CoV-2 was high for school students across studies internationally.

## Added value of this study

We investigated the number of daily SARS-CoV-2 infections among school students (aged 4–16 years), staff and their household members for over 18 months in Wales, UK. We found that vaccination uptake differed with much higher uptake in school staff compared with older students and household members. Poisson models with time-varying covariates suggested that less deprived students were more at risk for SARS-CoV-2, whereas less deprived school staff were at a lower risk. The Delta variant had a greater infection risk for students, but Omicron was a greater risk for staff and household members.

## Implications of all the available evidence

We present evidence that requires attention from public health and educational bodies. Further strategies need to recognise the protection vaccination affords, but more research is required on younger children. We show that sociodemographic factors are complexly associated with infection, and this should be further examined to determine the mechanisms. Lastly, more transmissible variants (i.e. Delta and Omicron) had greater associations with SARS-CoV-2 infection in school staff and students compared with their household members, meaning schools could be more vulnerable in the event of new, more transmissible variants.

## Declarations

### Guarantor

EL.

### Provenance

Not commissioned; peer reviewed by Azeem Majeed and Julie Morris.
