# Supplementary material for: Trends in SARS-CoV-2 infection and vaccination in school staff, students and their household members from 2020 to 2022 in Wales, UK: an electronic cohort study
Source: J R Soc Med. 2023 Jun 22;116(12):413–24. doi: 10.1177/01410768231181268 (PMC10767617; doi:10.1177/01410768231181268)
Supplement: sj-pdf-1-jrs-10.1177_01410768231181268 - Supplemental material for Trends in SARS-CoV-2 infection and vaccination in school staff, students and their household members from 2020 to 2022 in Wales, UK: an electronic cohort study [file sj-pdf-1-jrs-10.1177_01410768231181268.pdf]

Supplementary Material:

Figure S1: Crude rate of infections and PCR tests per 1,000 (rolling 7-day average) in the firebreak period for primary and younger middle (purple) and secondary and older middle school students (blue), school staff (pink), and household members (yellow). Dashed lines = rate of PCR tests, Solid fill = rate of positive PCR tests.

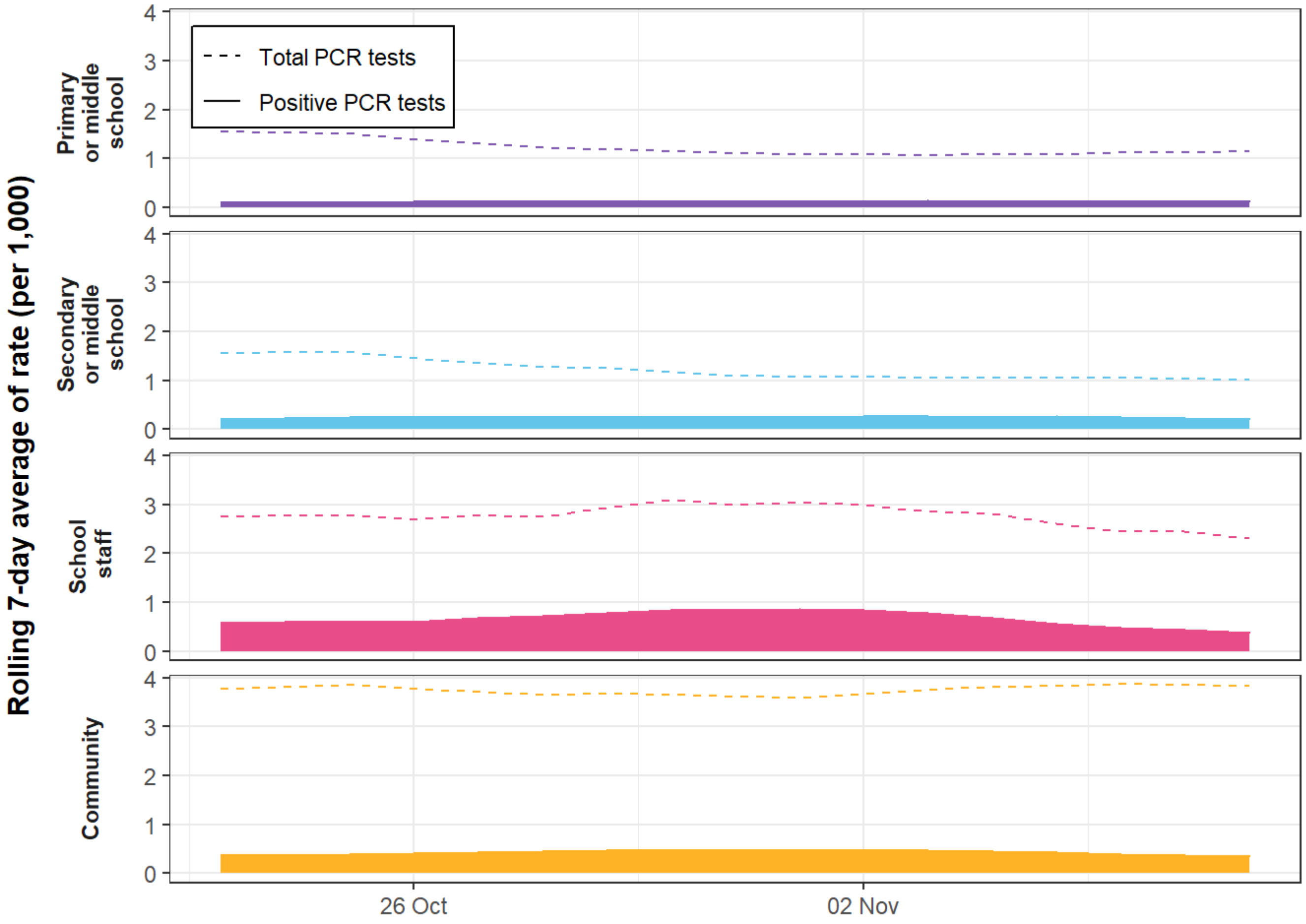

Figure S2: Crude rate of infections and PCR tests per 1,000 (rolling 7-day average) in the lockdown period for primary and younger middle (purple) and secondary and older middle school students (blue), school staff (pink), and household members (yellow). Dashed lines = rate of PCR tests, Solid fill = rate of positive PCR tests

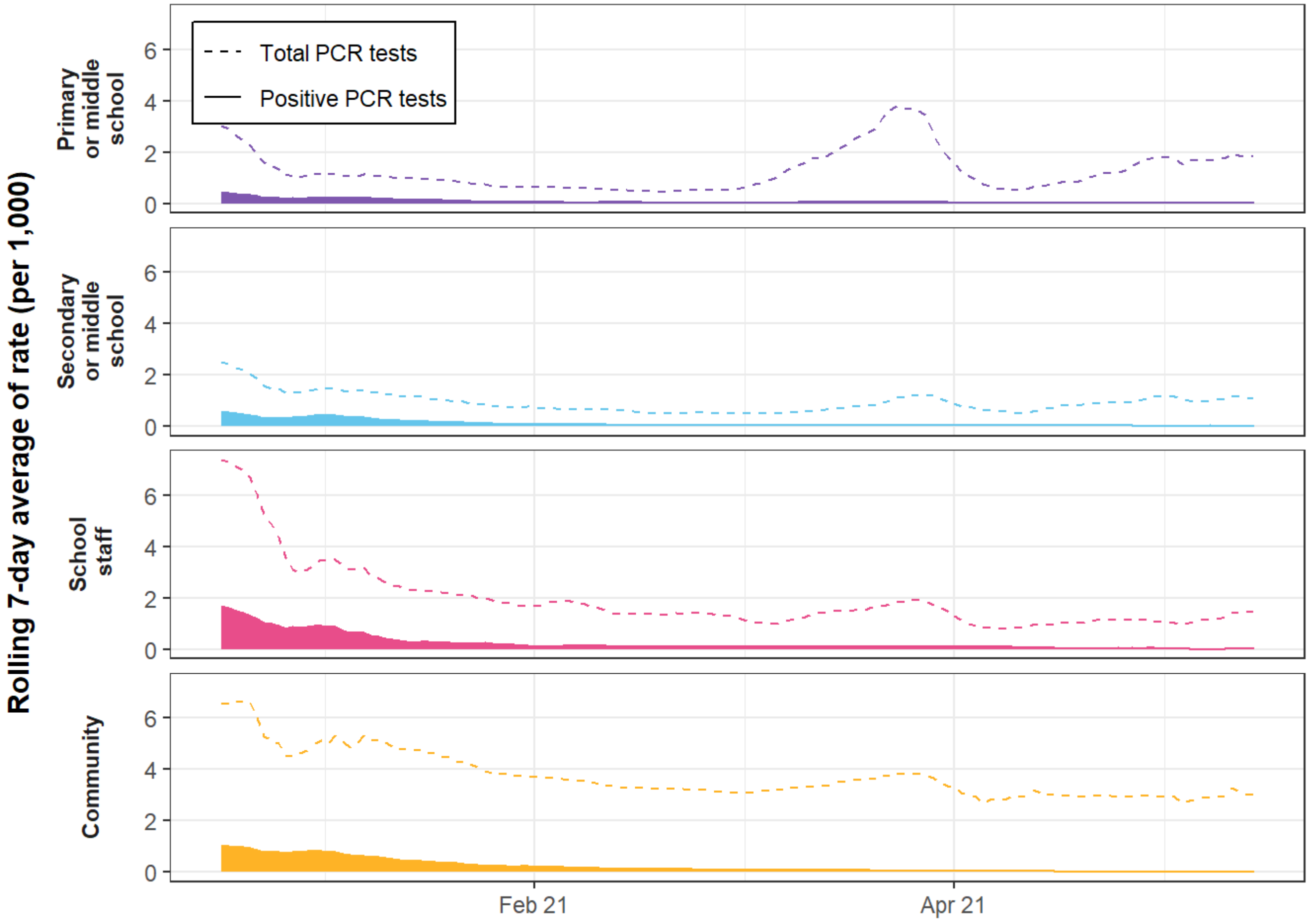

Table S1: Rate of PCR testing for each cohort

Type of cohort	WIMD	Total duration days	Total PCR	Rate per 1000 per year	Rate Ratio vs WIMD 5	95%CI
Primary School	1	33356592	98667	2.958	0.737	0.730, 0.744
Primary School	2	26886693	88701	3.299	0.822	0.814, 0.830
Primary School	3	23216526	82076	3.535	0.881	0.873, 0.889
Primary School	4	21944047	82792	3.773	0.94	0.931, 0.949
Primary School	5	21955804	88107	4.013	Ref	Ref
Primary School	NA	7807848	28201	3.612	0.9	0.889, 0.912
Secondary School	1	22269921	63279	2.841	0.823	0.814, 0.832
Secondary School	2	19262740	59909	3.110	0.9	0.890, 0.910
Secondary School	3	17474431	57268	3.277	0.949	0.938, 0.959
Secondary School	4	17579283	59666	3.394	0.983	0.972, 0.994
Secondary School	5	19081613	65912	3.454	Ref	Ref
Secondary School	NA	4375068	14123	3.228	0.935	0.918, 0.952
Staff	1	2729689	15103	5.533	1.073	1.053, 1.094
Staff	2	4181641	22159	5.299	1.028	1.011, 1.046
Staff	3	4858820	26588	5.472	1.062	1.045, 1.079
Staff	4	5585826	29989	5.369	1.042	1.025, 1.058
Staff	5	6586323	33950	5.155	Ref	Ref
Staff	NA	1400801	8184	5.842	1.133	1.106, 1.161
Household members	1	92317463	449237	4.866	1.116	1.111, 1.121
Household members	2	78816886	403673	5.122	1.174	1.169, 1.180
Household members	3	73659904	353106	4.794	1.1	1.094, 1.105
Household members	4	71611630	330971	4.622	1.06	1.055, 1.065
Household members	5	73705513	321413	4.361	Ref	Ref
Household members	NA	133011	565	4.248	0.974	0.897, 1.058
